# A Hybrid Soft Sensor Approach Combining Partial Least-Squares Regression and an Unscented Kalman Filter for State Estimation in Bioprocesses

**DOI:** 10.3390/bioengineering12060654

**Published:** 2025-06-15

**Authors:** Lucas Hermann, Andreas Kremling

**Affiliations:** Professorship for Systems Biotechnology, School of Engineering and Design, Technical University of Munich, Boltzmannstr. 15, 85748 Garching, Germany; lucas.hermann@tum.de

**Keywords:** soft sensor, coarse-grained modeling, partial least-squares regression, unscented Kalman filter

## Abstract

Real-time information on key state variables during fermentation is crucial for the effective optimization and control of bioprocesses. Specialized sensors for online or at-line monitoring of these variables are often associated with high costs, especially during early-stage process optimization. In this study, fed-batch processes of an L-phenylalanine (L-phe) production process were carried out using a recombinant *Escherichia coli* strain under varying inducer concentrations. The available online process variables from the L-phe production process were used to estimate the state variables biomass, glycerol, L-phe, acetate, and L-tyrosine (L-tyr) via partial least-squares regression (PLSR). These predictions were then incorporated as measurements into an unscented Kalman filter (UKF). The filter uses a coarse-grained model as a state estimator, which, in addition to extracellular variables, also provides information on intracellular states. The results of PLSR showed very good prediction accuracy for L-phe, moderate accuracy for glycerol, biomass, and L-tyr and poor performance for acetate concentrations. In combination with the UKF, the estimation of the L-phe concentrations was greatly improved compared to the CGM, whereas further improvement is still needed for the remaining state variables.

## 1. Introduction

Biotechnological processes offer a more sustainable alternative to petroleum-based processes. However, in order to be used industrially, they must be economically feasible [1]. This often requires numerous steps in strain and process optimization to achieve, for example, a desired yield or productivity. During the process optimization steps, samples have to be taken, usually manually, resulting in high labor input and cost, especially for long and complex processes. Also, as a result, important process information in longer processes is often lost, for instance, due to missing sample collections during the night [2]. Moreover, typically only extracellular concentrations in the reactor are measured, while intracellular process states remain unconsidered. One way to gain additional process information is through so-called soft sensors. Soft sensors combine measurement data from physical sensors with a model to predict unknown process variables and have been used in bioprocesses for over 20 years [3,4,5,6]. Based on the amount of process understanding included in the model, it can be classified as either data-driven, knowledge-based, or a combination of both (hybrid) [7].

Data-driven soft sensors allow the prediction of unknown variables, even without detailed mechanistic knowledge, by only applying a black-box model. These models are easy to implement but require large amounts of high-quality training data. While they often fit the training data well, their prediction performance on unknown processes is often limited due to biological variability and process modifications [8]. Various modeling techniques are available to implement data-driven soft sensors, such as principal component regression, support vector regression (SVR), artificial neural networks (ANNs), and partial least-squares regression (PLSR) [7]. In PLSR, the original input and output variables are transformed into latent variables (LVs), thereby reducing the dimensionality of the dataset. A linear relationship is then identified by maximizing the covariance between the projections of the input and output variables [9]. Typical input variables for data-driven approaches include online available process parameters such as stirrer speed, temperature, pH, dissolved oxygen (pO_2_), off-gas CO_2_/O_2_, and flow rates [10,11]. In addition, advanced online and at-line measurement techniques based on various spectroscopic methods, such as Raman spectroscopy, near-infrared spectroscopy (NIR), and 2D fluorescence, can be integrated [12,13,14,15].

If more knowledge about the functional relations in the process is available, a knowledge-based soft sensor can be implemented. Those mechanistic (white-box) models are derived based on the physical and chemical properties of the system. They are often more complex to implement but provide more explainable information about the process and require less data [16]. The Kalman filter (KF) is a knowledge-based soft sensor. It makes a prediction based on a mathematical model, which is then corrected in the next step using sensor or measurement data. However, the classical KF is only applicable to linear processes. To model nonlinear processes, the extended Kalman filter (EKF) can be used, which approximates the state transition and measurement functions at each measurement step via a first-order Taylor expansion. For highly nonlinear models, the unscented Kalman filter (UKF) provides a better approximation. The UKF generates sigma points that can be directly propagated through the nonlinear system, allowing nonlinearities to be captured more accurately [13,17].

In hybrid modeling, data-driven and knowledge-based approaches are combined to overcome the limitations and exploit the benefits of each method. For example, when training data is limited or costly, knowledge-based models can generate data to support training, reducing the need for extensive experiments. By incorporating mechanistic knowledge, hybrid models handle noise and nonlinearities more effectively than data-driven models alone. Additionally, when mechanistic understanding is incomplete, a hybrid model can fill these gaps, resulting in a more accurate and robust model. In summary, the advantages of this approach include greater transparency, a broader knowledge base, and more cost-effective model development [18].

To implement a KF, an accurate process model that sufficiently describes the system dynamics is necessary. However, developing such a model is often challenging for complex biological processes. One approach to address this is the use of coarse-grained models (CGMs). These models abstract cellular processes by grouping functionally related components into a reduced number of subsystems [19,20]. A key aspect of CGMs is resource allocation, which assumes that cells distribute their available resources, like nutrients and proteins, for optimized growth [21]. By incorporating this approach, it becomes possible to represent the mass fractions of different protein groups relative to the total biomass of the cell. This, in turn, enables the coupling of biochemical reactions to the corresponding proteome groups, making them dependent on the underlying resource distribution. This approach reduces system complexity while still providing a sufficient description of cellular processes.

In biotechnological processes, the proteome often plays a crucial role. The desired product is often a protein itself or requires enzymes for its biosynthesis. Due to the spatial limitations within a cell, the expression of heterologous proteins necessitates a redistribution of the cellular proteome [22]. For instance, Basan et al. [23] demonstrated that the expression of heterologous proteins can lead to acetate formation even at low growth rates. The observed formation of the unwanted byproduct acetate can be explained by a more proteome-efficient formation via fermentation compared to respiration. This often-observed phenomenon of overflow metabolism can be described using CGMs, making them an interesting tool for optimizing heterologous protein expression to enhance product synthesis and minimize unwanted byproduct formation.

**Figure 1:**
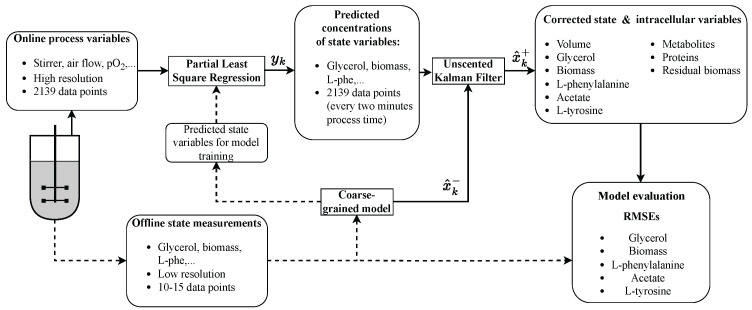
Process flowchart of the implemented hybrid approach using a CGM in combination with a UKF and PLSR. In the first step, online process data from a bioreactor are used to estimate the concentrations of key state variables via PLSR. These estimates are then passed to a UKF as the measurement vector $y_{k}$. The CGM predicts the a priori state ${\hat{x}}_{k}^{-}$, which is then updated in the UKF with the measurements from the PLSR to obtain the corrected state estimate ${\hat{x}}_{k}^{+}$. This corrected state includes both the extracellular concentrations in the reactor and the intracellular variables. The dotted arrows indicate that the CGM was also used during the initial training phase of the PLSR models, in which offline measurement data were used to fit the model, ensuring that a sufficient number of response-variable data points were available for training.

This study investigates the applicability of a CGM within a hybrid approach combining a UKF and PLS model to describe an L-phenylalanine (L-phe) production process using a recombinant *Escherichia coli* strain. The process consists of three different phases: an initial batch phase, followed by a biomass production phase, and finally an L-phe production phase, in which the cells are induced with Isopropyl β-D-1-thiogalactopyranoside (IPTG). Three experiments are conducted using different inducer concentrations. The first experiment is carried out with 0.3 mM IPTG (process 1), while for the other two, a lower concentration of 0.01 mM IPTG (processes 2 and 3) is used. In the first step, PLS models are trained using standard online process inputs, such as stirrer speed, aeration rate, pO_2_, temperature, pH, CO_2_ concentration in the off-gas, substrate feed rate (mass flow), and base addition, to predict key process variables, including biomass, glycerol, L-phe, acetate, and L-tyrosine (L-tyr) concentration, using the data from process 1. The trained models are then validated on two new processes (processes 2 and 3) and their predictions compared to offline measurements. Subsequently, the predictions from the PLS models are integrated into a UKF as measurements, while the system states are estimated using the CGM. In addition to capturing extracellular variables such as volume, biomass, glycerol, L-phe, acetate, and L-tyr, the CGM also estimates intracellular states, including protein, metabolite, and residual biomass concentrations. Measurement and model uncertainties are determined using the data from process 2, and the complete hybrid model is subsequently validated on an unseen dataset (process 3). The complete workflow can be seen in Figure 1.

## 2. Materials and Methods

### 2.1. Strain

For the L-phenylalanine production process, the recombinant *E. coli* strain FUS4 (pF81_kan_) was used [24]. The FUS4 strain is a derivative of the *E. coli* K-12 W3110 strain with chromosomal deletions of the genes *pheA*, *aroF*, and *tyrA*, making it a double auxotroph mutant for L-phe and L-tyr. To produce L-phe, the strain harbors the pF81 plasmid, which carries the genes *aroF*, *pheA’*, *aroB*, and *aroL* under an inducible $P_{tac}$-promoter. The strain used in this study also carries four fluorescent reporter proteins, with three integrated into the chromosome and one encoded on the plasmid, which are not further considered in this work. Further information about the fluorescent proteins can be found in Hoang et al. [25].

### 2.2. Media Composition

A minimal medium with glycerol as the sole carbon source was used for cultivation, using the composition and concentrations described by Weiner et al. [24].

### 2.3. Preculture

Cells of *E. coli* FUS4 (pF81_kan_) were streaked from a cryo vial onto a minimal medium agar plate containing glycerol as the sole carbon source and incubated at 37 °C for at least 66 h. A single colony was then picked and inoculated into a 100 mL shake flask containing 10 mL of minimal medium supplemented with 7 g L^−1^ glycerol. The culture was incubated for 24 h at 37 °C and 150 rpm in a rotary shaker (MaxQ 8000 Stackable Incubator, Thermo Fisher Scientific, Waltham, MA, USA). After 24 h, the optical density at 600 nm (OD_600_) was measured (Genesys 10UV, Thermo Fisher Scientific, Waltham, MA, USA), and a defined volume was used to inoculate a second preculture in a 500 mL shake flask containing 100 mL of minimal medium to a starting OD_600_ of 0.01. The second preculture was incubated for at least 24 h at 37 °C and 250 rpm. After 24 h, the cultures were centrifuged (Heraeus Megafuge 16R Centrifuge, Thermo Fisher Scientific, Waltham, MA, USA) at 3200× *g* for 10 min. The supernatant was discarded, and the cell pellets were resuspended in a minimal medium without glycerol and amino acids. The resuspended cells were then used to inoculate the bioreactor to a starting OD_600_ of 0.05.

### 2.4. Bioreactor

L-phe production was carried out as a fed-batch process in a 3.6 L stirred-tank bioreactor (Labfors 5, Infors GmbH, Bottmingen, Switzerland). The reactor was equipped with two six-blade Rushton turbines and three baffles. Prior to inoculation, sterile minimal medium containing 4 g L^−1^ glycerol was pumped into the bioreactor to a starting volume of 1 L. Throughout the process, the temperature was maintained at 37 °C, and the pH was monitored using a two-point calibrated pH probe (EasyFerm Plus PHI Arc 325, Hamilton Bonaduz AG, Bonaduz, Switzerland) and maintained at pH 7 by the addition of 42% phosphoric acid and 25% ammonia. pO_2_ was measured using a single-point calibrated pO_2_ probe (VisiFerm DO Arc 325 H0, Hamilton Bonaduz AG, Bonaduz, Switzerland) and kept above 40% by increasing the stirrer speed (up to a maximum of 1200 rpm) or the aeration rate (up to 5 L/min). Foam formation was controlled using an antifoam probe and regulated by the addition of an antifoam solution (AF204, Sigma-Aldrich, Taufkirchen, Germany). Off-gas oxygen (O_2_) and carbon dioxide (CO_2_) concentrations were monitored online using a gas analyzer (BlueInOne Ferm, BlueSens, Herten, Germany). The process was adapted and slightly modified from the protocol described by Weiner et al. [24] and consisted of three distinct phases. It began with an initial batch phase, which ended with the depletion of glycerol, indicated by a characteristic pO_2_ peak. Subsequently, the biomass production phase was initiated, in which a specific growth rate of $\mu_{set}$ = 0.1 h^−1^ was maintained. Feeding during this phase was carried out using a feed medium containing 312.5 g L^−1^ glycerol, 1.65 g L^−1^ L-phe, 3.75 g L^−1^ L-tyr, 40 g L^−1^ ammonium sulfate, and 0.1 g L^−1^ kanamycin. To fully dissolve the L-tyr, the feed medium was titrated with 5 M potassium hydroxide. After a minimum of 21 h in the biomass production phase, induction was carried out using either 0.3 mM (process 1) or 0.01 mM IPTG (processes 2 and 3). Simultaneously, a second feed medium was added at a rate of 0.18 $g_{\mathrm{glycerol}}$ $g_{\mathrm{biomass}}^{-1}$ $h^{-1}$. This second feed consisted of 800 g L^−1^ glycerol, 8 g L^−1^ ammonium sulfate, 8 g L^−1^ ammonium phosphate, and 0.1 g L^−1^ kanamycin. Additionally, 6.75 mL of four times concentrated minimal medium (without glycerol or amino acids) was added at the beginning of the biomass production phase, and 13.5 mL of the same medium was added at the start of the production phase.

### 2.5. Offline Analytics

For determining the biomass, 2 mL of cell suspension was centrifuged at 21,130× *g* at 4 °C for 20 min in dried, pre-weighed (80 °C for at least 24 h) 2 mL centrifuge tubes. The supernatant was filtered (pore size 0.6 μm) and stored at 4 °C until the determination of extracellular metabolites by high-performance liquid chromatography (HPLC). After the pellet was dried again for 24 h, the tube was weighed again, and the difference was calculated based on the change in weight. The organic compounds (glycerol and acetate) were quantified using an HPLC (Prominence-i LC-2030C, Shimadzu, Kyoto, Japan) equipped with an ion-exchange column (Aminex HPX-87H 300 mm × 7.8 mm, Bio-Rad, Hercules, CA, USA) and a refractive index detector (RID-20A, Shimadzu, Kyoto, Japan). 10 μL of sample was measured with an isocratic profile using a 5 mM sulfuric acid solution at 0.6 mL min^−1^ and 60 °C for 30 min. The amino acids L-phe and L-tyr were also quantified using the same HPLC system, with a sample injection volume of 1 μL. A detailed protocol for the amino acid determination can be found in [25].

### 2.6. Coarse-Grained Model

To describe the L-phe production process, a CGM was used. In the model, originally described by Doan et al. [20], reaction rates depend on proteome fractions, which are grouped according to their cellular function. The proteome fractions are, therefore, transport and catabolism ($\phi_{T}$), ribosomes ($\phi_{R}$), remaining proteins ($\phi_{Q}$), and L-phe production proteins ($\phi_{F_{p}}$). Besides the states in the bioreactor, namely volume, glycerol, biomass, L-phe, acetate, and L-tyr (since the strain is L-tyrosine auxotroph), the model also enables the investigation of intracellular variables such as metabolites, proteins, and remaining biomass. A detailed description of the adapted model, parameter estimation, confidence intervals, and error variance can be found in the Supplementary Materials.

### 2.7. PLSR

PLSR was used to model the relationship between the online process variables and the offline state variables. The online input variables included stirrer speed, aeration rate, pO_2_ concentration, temperature, pH, CO_2_ concentration in the off-gas, substrate feed rate (mass flow), and base addition. These inputs were correlated with offline measurements of the biomass, glycerol, L-phe, acetate, and L-tyr concentrations. To train the PLS model, input and output data must be available for each time point. In biotechnological processes, however, offline measurements are typically available only at irregular intervals, as they require manual and time-consuming sampling. To overcome this limitation and allow for continuous model training, the output variables were generated using a previously validated CGM that had already been successfully applied to this process. The simulated outputs were then linearly interpolated to align with the time points of the online input data. Given that the process comprises three distinct process phases with different dynamics, separate PLS models were constructed for each phase. The MATLAB built-in function *plsregress*, which uses the SIMPLS algorithm [26], was used for output variable reduction and parameter estimation. To ensure consistent starting conditions across each phase, all input data were mean-centered relative to their values at the start of each respective phase *p*.

(1) $${\tilde{X}}_{i,p}=X_{i,p}-X_{0,p}\;i=1,\ldots ,n\;p=1-3$$

where ${\tilde{X}}_{i,p}$ is the mean-centered input value at time point *i*, $X_{i,p}$ is the original input value at time point *i*, and $X_{0,p}$ is the input value at the beginning of phase *p*.

Model training was performed using data from process 1, in which induction with 0.3 mM IPTG was applied during the production phase. This is referred to as the reference process. Model validation was carried out using five-fold cross-validation, with the optimal number of LVs determined based on the root mean square error of cross-validation (RMSECV). Model performance was subsequently tested on data from processes 2 and 3, in which a lower inducer concentration (0.01 mM) during the production phase was used. Model accuracy was quantified using the root mean square error (RMSE), calculated based on the interpolated output values for the training data and experimentally measured values for the tested datasets.

### 2.8. Unscented Kalman Filter

The UKF was implemented as described in [27,28], with additive process and measurement noise incorporated into the nonlinear system, which is described as follows:

(2) $$\begin{array}{cc} \; & x_{k}=f\left(x_{k-1},u_{k-1}\right)+w_{k-1} \end{array}$$

(3) $$\begin{array}{cc} \; & y_{k}=h\left(x_{k}\right)+v_{k} \end{array}$$

(4) $$\begin{array}{cc} \; & w_{k}∼N\left(0,Q\right) \end{array}$$

(5) $$\begin{array}{cc} \; & v_{k}∼N\left(0,R\right) \end{array}$$

Here, $x_{k}$ denotes the system state at time step *k*, $y_{k}$ is the corresponding measurement, $u_{k}$ is the control input, and $w_{k}$ and $v_{k}$ represent process and measurement noise, respectively. At time step k=0, the UKF was initialized with the initial state estimate and error covariance as follows:

(6) $$\begin{array}{cc} \; & {\hat{x}}_{0}^{+}=E\left[x_{0}\right] \end{array}$$

(7) $$\begin{array}{cc} \; & P_{0}^{+}=E\left[\left(x_{0}-{\hat{x}}_{0}^{+}\right){\left(x_{0}-{\hat{x}}_{0}^{+}\right)}^{⊤}\right] \end{array}$$

For the prediction step from time k−1 to *k*, the sigma points were generated.

(8) $$\begin{array}{cccc} \; & \chi_{k-1}^{\left(0\right)}={\hat{x}}_{k-1}^{+} & \; & \end{array}$$

(9) $$\begin{array}{cccc} \; & \chi_{k-1}^{\left(i\right)}={\hat{x}}_{k-1}^{+}+{\tilde{x}}^{\left(i\right)}\; & \; & i=1,\ldots ,2n \end{array}$$

(10) $$\begin{array}{cccc} \; & {\tilde{x}}^{\left(i\right)}={\left(\sqrt{\left(n+\lambda \right)\;P_{k-1}^{+}}\right)}_{i}^{⊤}\; & \; & i=1,\ldots ,n \end{array}$$

(11) $$\begin{array}{cccc} \; & {\tilde{x}}^{\left(n+i\right)}=-{\left(\sqrt{\left(n+\lambda \right)\;P_{k-1}^{+}}\right)}_{i}^{⊤}\; & \; & i=1,\ldots ,n \end{array}$$

Here, *n* denotes the size of the state vector, and λ is a scaling parameter that determines the spread of the sigma points around the mean. Here it is expressed as

(12) $$\lambda =\alpha^{2}\left(n+\kappa \right)-n$$

The parameters α and κ are tuning parameters that influence the spread of the sigma points around the mean. Specifically, α controls the overall spread, while κ is a secondary scaling parameter. The square root of the scaled covariance matrix was computed using the Cholesky factorization. The resulting sigma points were then propagated through the nonlinear system f(·):

(13) $$\chi_{k}^{\left(i\right)}=f\left(\chi_{k-1}^{\left(i\right)},u_{k}\right)$$

Subsequently, the propagated sigma points were combined to compute the a priori state estimate at time step *k*, as well as the a priori error covariance, with the additive process noise *Q*.

(14) $${\hat{x}}_{k}^{-}=\sum_{i=0}^{2n}W_{m}^{\left(i\right)}\chi_{k}^{\left(i\right)}$$

(15) $${\hat{P}}_{k}^{-}=\sum_{i=0}^{2n}W_{c}^{\left(i\right)}\left(\chi_{k}^{\left(i\right)}-{\hat{x}}_{k}^{-}\right){\left(\chi_{k}^{\left(i\right)}-{\hat{x}}_{k}^{-}\right)}^{T}+Q$$

The weights for the mean $W_{m}$ and covariance $W_{c}$ are given by

(16) $$\begin{array}{cc} \; & W_{m}^{\left(0\right)}=\frac{\lambda }{n+\lambda } \end{array}$$

(17) $$\begin{array}{cc} \; & W_{c}^{\left(0\right)}=\frac{\lambda }{n+\lambda }+\left(1-\alpha^{2}+\beta \right) \end{array}$$

(18) $$\begin{array}{cccc} \; & W_{m}^{\left(i\right)}=W_{c}^{\left(i\right)}=\frac{\lambda }{2(n+\lambda )}\; & \; & i=1,\ldots ,2n \end{array}$$

where the parameter β improves the approximation by accounting for the higher-order statistical properties of the state distribution (for Gaussian distributions, β=2 is considered optimal).

If a measurement was available at time step *k*, the correction step was carried out. For this purpose, the sigma points were propagated through the measurement function h(·):

(19) $$\gamma_{k}^{\left(i\right)}=h\left(\chi_{k}^{\left(i\right)}\right)$$

The propagated sigma points were then again weighted and combined to obtain the predicted measurement ${\hat{y}}_{k}$ and its associated covariance $P_{y}$ at time step *k*.

(20) $${\hat{y}}_{k}=\sum_{i=0}^{2n}W_{m}^{\left(i\right)}\gamma_{k}^{\left(i\right)}$$

(21) $$P_{y}=\sum_{i=0}^{2n}W_{c}^{\left(i\right)}\left(\gamma_{k}^{\left(i\right)}-{\hat{y}}_{k}\right){\left(\gamma_{k}^{\left(i\right)}-{\hat{y}}_{k}\right)}^{T}+R$$

The cross-covariance between the predicted state and the predicted measurement was then computed as

(22) $$P_{xy}=\sum_{i=0}^{2n}W_{c}^{\left(i\right)}\left(\chi_{k}^{\left(i\right)}-{\hat{x}}_{k}^{-}\right){\left(\gamma_{k}^{\left(i\right)}-{\hat{y}}_{k}\right)}^{T}$$

and based on this, the Kalman gain was calculated.

(23) $$K_{k}=P_{xy}P_{y}^{-1}$$

Finally, the updated state estimate and its error covariance were obtained as

(24) $$\begin{array}{cc} \; & {\hat{x}}_{k}^{+}={\hat{x}}_{k}^{-}+K_{k}\left(y_{k}-{\hat{y}}_{k}\right) \end{array}$$

(25) $$\begin{array}{cc} \; & P_{k}^{+}=P_{k}^{-}-K_{k}P_{y}K_{k}^{T} \end{array}$$

To ensure that the updated state variables did not contain negative concentrations, an additional correction step was applied, as described in Kraemer and King [13]. If any state variable exceeded its lower bound after the update, a new set of sigma points was generated using the updated state estimate ${\hat{x}}_{k}^{+}$ and covariance $P_{k}^{+}$. Sigma points that did not satisfy the constraint were projected onto the constraint boundary, and with the new set of sigma points, another prediction step was carried out as described above.

The measurement noise covariance matrix *R* was constructed using the squared RMSE values of the PLSR-based state estimates from process 2 as the diagonal entries, as described in Kraemer and King [13]. The diagonal entries in the process noise covariance *Q* were determined by solving a least-squares optimization problem, minimizing the mean squared error (MSE) between the updated state estimates and the offline measurements using the function *patternsearch* in MATLAB, similar to the approach proposed in Narayanan et al. [29]. All simulations were performed using MATLAB Version 24.1.0.2628055 (R2024a) Update 4.

In the hybrid approach, the online process variables were used as inputs for the PLS models to estimate the state variables biomass, glycerol, L-phe, acetate, and L-tyr. These estimates were then used as the measurement vector $y_{k}$ in the UKF, thereby enabling correction of the model-based state prediction ${\hat{x}}_{k}^{-}$ calculated by the CGM. Prediction steps were performed every 60 s, and measurement updates were available every 120 s. A detailed overview of the workflow is shown in Figure 1.

## 3. Results

### 3.1. PLSR

The PLS models for biomass, glycerol, L-phe, acetate, and L-tyr were generated using online process data in combination with simulated offline state variables. Process data were recorded every 120 s, resulting in a total of 2139 data points per input and output variable in the training dataset. Given that the process is divided into three distinct phases, where it is assumed that each phase exhibits different dynamics, a separate model was trained for each phase. For training and validation, data from the reference process (process 1), in which the cells were induced with 0.3 mM IPTG during the production phase, were used. Model performance was then tested on separate prediction datasets derived from processes 2 and 3, in which only 0.01 mM IPTG was applied during the production phase. To determine the optimal number of LVs and reduce the risk of overfitting, the models were evaluated using k-fold cross-validation with *k* = 5. This resulted in three LVs for phase 1, five LVs for phase 2, and two LVs for phase 3. The results of the cross-validation can be seen in Figure S4 in the Supplementary Materials.

**Figure 2:**
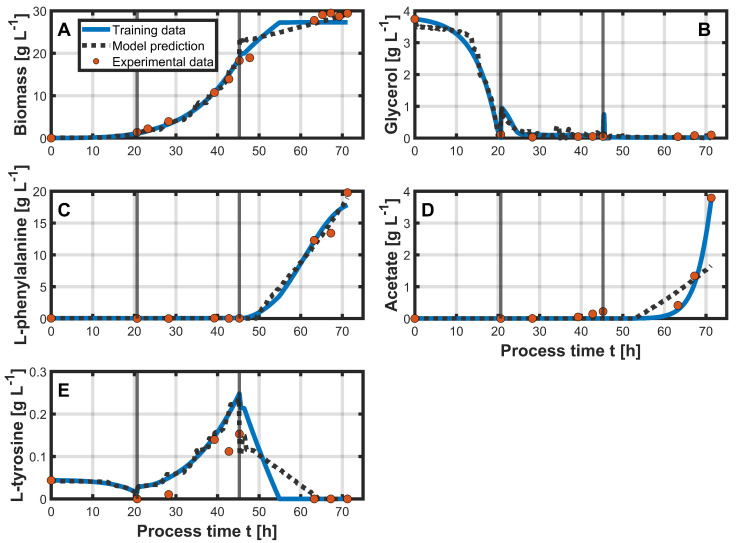
Training of the PLS models on the data from process 1 using online process variables as inputs. Vertical gray lines indicate the different process phases. The model predictions for the state variables (biomass (**A**), glycerol (**B**), L-phe (**C**), acetate (**D**), and L-tyr (**E**)) are shown as black dotted lines over process time. The training data for these state variables, generated using the CGM, are represented by solid blue lines. Orange dots indicate the corresponding offline measurements.

The results of the PLS models are presented in Figure 2. The calculated RMSEs can be found in Table 1. The PLS model-predicted biomass demonstrated good agreement with the training data, with an RMSE of 0.95 g L^−1^. Up to approximately 45 h of process time, the prediction aligned well with the training data. However, at the onset of the production phase, the agreement slightly decreased (Figure 2A). Since the training data were fitted to the experimental data, it is difficult to evaluate the actual biomass trend during this period due to a lack of experimental data between 50 and 60 h of process time. The model also predicted L-phe concentrations with high accuracy (Figure 2C), achieving an RMSE of 0.43 g L^−1^. Similarly, prediction of the glycerol concentrations also showed good agreement (Figure 2B), with an RMSE of 0.12 g L^−1^. In contrast, for the concentrations of acetate, the PLS models did not perform as well during the production phase. While the onset of acetate production was experimentally observed at around 60 h, the model predicted an earlier onset at approximately 50 h, and the final predicted concentration distinctly underestimated both the experimental and training data (Figure 2D). The RMSE in this case was 0.3 g L^−1^. The prediction of L-tyr concentrations is shown in Figure 2E, with good agreement up to the production phase, after which model accuracy declined from around 45 h onward, resulting in an RMSE of 0.02 g L^−1^.

**Figure 3:**
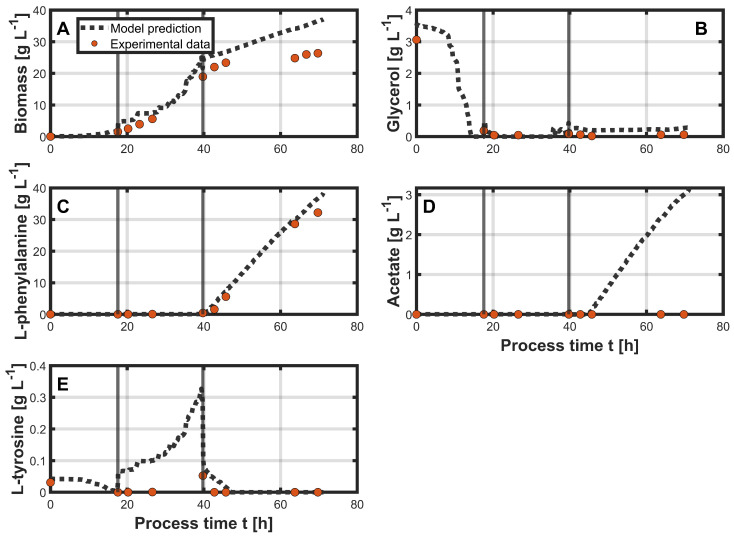
Predictions using the trained PLS models on the unseen data from process 2 with online process variables as inputs. Vertical gray lines indicate the different process phases. Model predictions for the state variables (biomass (**A**), glycerol (**B**), L-phe (**C**), acetate (**D**), and L-tyr (**E**)) are shown as black dotted lines over process time. Orange dots represent the corresponding offline measurements.

The PLS models generated in the previous step were subsequently applied to the prediction dataset. In the following, only process 2 is presented and discussed in detail, as illustrated in Figure 3. Process 3 is provided in the Supplementary Materials (Figure S7), as both processes exhibited similar model behavior. The biomass concentration, shown in Figure 3A, was consistently slightly overestimated throughout the entire process. This overestimation increased with rising biomass concentration over time and is reflected in the resulting RMSE of 5.59 g L^−1^. Furthermore, the measured biomass concentrations were slightly lower than in the reference process. For the glycerol concentrations (Figure 3B), the model initially showed a slight overestimation. It also predicted complete depletion at around 14.5 h of process time, approximately three hours before glycerol depletion was experimentally confirmed. For the remaining process, the glycerol concentration remained close to 0 g L^−1^, which was accurately captured by the model, resulting in an RMSE of 0.26 g L^−1^. L-phe (Figure 3C) remained at nearly 0 g L^−1^ during the growth phase and began to accumulate during the production phase, marked by the second gray vertical line. The measured concentration reached over 30 g L^−1^ by the end of the process, which was approximately 10 g L^−1^ higher than in the reference process. This increase was well captured by the model, resulting in an RMSE of 1.81 g L^−1^. Interestingly, no acetate formation (Figure 3D) was observed experimentally during the entire process. In contrast, the model predicted the onset of acetate accumulation at around 45 h, reaching a final concentration of approximately 3 g L^−1^ at the end of the process. This discrepancy resulted in an RMSE of 1.29 g L^−1^. For L-tyr concentrations (Figure 3E), the initial concentration was accurately predicted by the model. However, during the second phase of the process (first gray vertical line), the concentration was consistently overestimated and remained above the measured values at all time points. Nevertheless, the model adequately captured the declining trend during the production phase, resulting in an RMSE of 0.09 g L^−1^.

In summary, it was observed that the PLS models experienced particular difficulty in accurately estimating biomass and acetate concentrations during the production phase, as well as L-tyr concentrations during the biomass growth phase. As PLSR depends on correlations between online process variables and system state variables, reliable prediction is only possible if changes in the state variables are followed by corresponding variations in the online process variables. However, many process parameters, such as temperature and pH, remained constant throughout the fermentation, aside from minor deviations within the setpoint tolerance. Particularly during the production phase, most online variables remained nearly unchanged (Figures S3–S5). Due to the constant feed rate, oxygen uptake also remained stable, which, in turn, led to steady agitation speed, aeration rate, and pO_2_ levels. As a result, off-gas CO_2_ concentrations also showed minimal variations. The only process variable that changed notably during the production phase was base addition. L-phe is an ampholyte [30], slightly lowering the pH and thus requiring the addition of base to maintain the setpoint of pH 7. This correlation explains the relatively good performance of the PLS models in predicting the L-phe concentrations. Acetate, in contrast, caused a more pronounced drop in pH, leading to increased base consumption. This was reflected in a subtle increase in the slope of the base addition curve (Figure S5H). However, since acetate was only produced in small amounts toward the end of the process, the resulting change appeared insufficient in the input variables to allow the PLS models to accurately capture acetate formation. Another crucial factor for building a reliable prediction model is the availability of high-quality training data. For long, multi-phase bioprocesses, a large number of offline measurements are typically required to generate a representative dataset. Alternatively, as done in this study, a model can be used to simulate additional data points. While this approach enables model training with fewer experimental measurements, it also introduces a dependency on the accuracy of the underlying model, which may lead to discrepancies if the model does not predict the biological data correctly.

### 3.2. Hybrid Approach

The UKF was implemented using the CGM as the state estimator and the predictions from the PLS models, which were trained on process 1, as measurement inputs. As online process data were recorded every 120 s, the correction step in the UKF was performed at that interval, while the prediction step was performed for a time interval of 60 s. The measurement noise covariance matrix *R* was defined using the MSE of the PLSR predictions from process 2 as the diagonal entries. The process noise covariance matrix *Q* was estimated by minimizing the MSE between the hybrid UKF predictions and the offline measurements of process 2 (see Materials and Methods). The resulting hybrid approach was then applied to the unseen data from process 3 using the same set of parameters. To evaluate model performance, the RMSE was calculated between the offline measurements and the interpolated predictions from the UKF and the CGM, as shown in Table 2.

**Figure 4:**
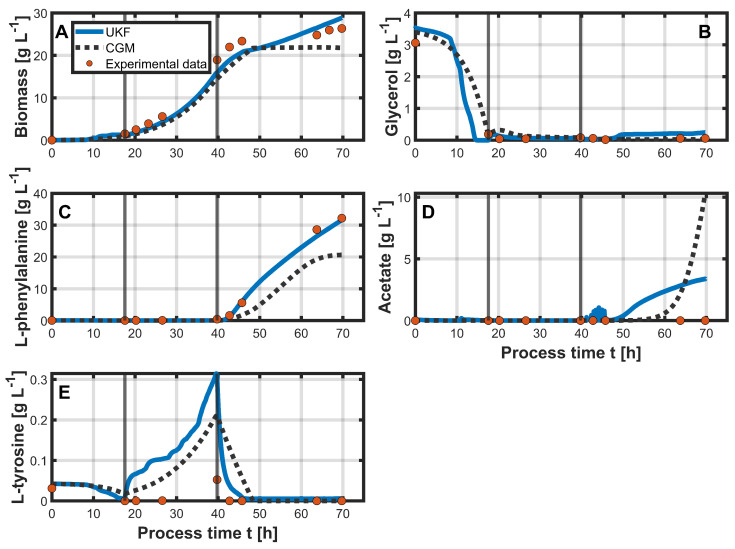
Simulation of process 2 using the UKF with the CGM as the state estimator and measurements from PLSR. The UKF predictions are shown as blue solid lines, while the CGM predictions are represented by black dotted lines for the state variables: biomass (**A**), glycerol (**B**), L-phe (**C**), acetate (**D**), and L-tyr (**E**). Vertical gray lines indicate the different process phases. Orange dots represent the corresponding offline measurements. The offline measurement data were used to estimate the diagonal entries in the process noise covariance matrix *Q*.

The results of the hybrid approach can be seen in Figure 4, which shows the state variables: biomass (Figure 4A), glycerol (Figure 4B), L-phe (Figure 4C), acetate (Figure 4D), and L-tyr (Figure 4E). The initial values for the state variables were identical in both approaches. For glycerol, the UKF predicted a distinctly faster decrease in concentrations. Similar to the PLSR estimation, the glycerol concentrations were already predicted to reach 0 g L^−1^ approximately three hours before glycerol depletion was experimentally confirmed. For most of the process time, the UKF estimated glycerol concentrations close to 0 g L^−1^. However, during the production phase, a slight increase to approximately 0.25 g L^−1^ was observed. The CGM, in contrast, accurately captured the end of the batch phase and maintained a glycerol concentration close to 0 g L^−1^ throughout the process, closely aligning with the offline measurements. The UKF consistently estimated higher biomass concentrations than the CGM. Up to approximately 48 h of process time, the UKF estimates lay just below the actual measurements and outperformed the CGM estimates. Toward the end of the process, the UKF predicted a slight increase in biomass, which resulted in an overestimation of the process variable, while the CGM predicted almost constant biomass concentrations during the production phase. L-tyr was completely depleted during the batch phase, a trend that was not fully captured by the CGM, while the UKF provided a slightly better approximation. In the second process phase, both models overestimated the L-tyr concentration, with the UKF deviating more strongly, predicting a concentration of 0.3 g L^−1^ at the end of the phase, compared to the measured value of approximately 0.05 g L^−1^. Nevertheless, the subsequent decline in the production phase was better captured by the UKF, although the measured concentration reached 0 g L^−1^ earlier than predicted. The concentration of L-phe was well approximated by the UKF. In the first two phases, no increase was measured, which was correctly reflected by both models. Upon the induction and start of the production phase, a rapid increase in L-phe was measured, which was closely approximated by the UKF. The CGM, however, predicted a noticeably slower increase. The maximum measured concentration of approximately 33 g L^−1^ was also accurately estimated by the UKF, while the CGM predicted a lower maximum of around 20 g L^−1^ near the end of the process. Acetate concentration was poorly predicted by the UKF and the CGM. The measured acetate concentrations remained near 0 g L^−1^ throughout the process. In contrast, the UKF predicted an increase starting at around 50 h, reaching just under 4 g L^−1^ by the end of the process, while the CGM predicted an increase beginning at approximately 55 h, ending in a final concentration of around 10 g L^−1^.

**Figure 5:**
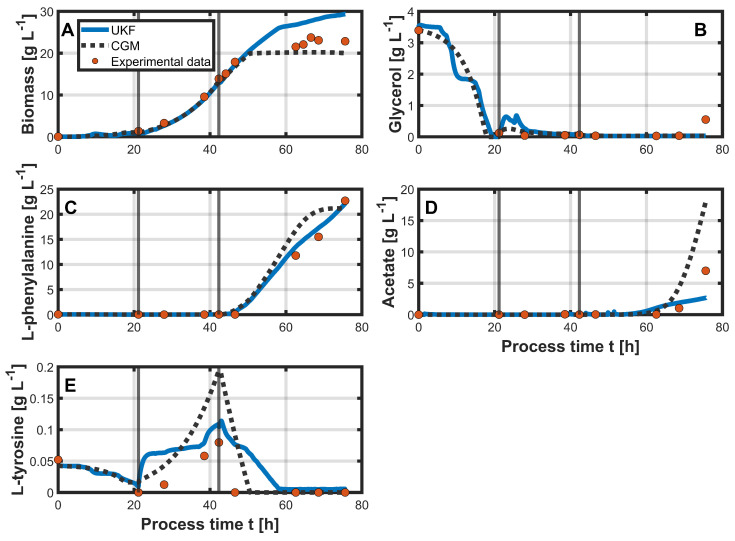
Simulation of process 3 using the UKF with the CGM as the state estimator and measurements from PLSR. All parameters were transferred from the simulation of process 2. The UKF predictions are shown as blue solid lines, while the CGM predictions are represented by black dotted lines for the state variables: biomass (**A**), glycerol (**B**), L-phe (**C**), acetate (**D**), and L-tyr (**E**). Vertical gray lines indicate the different process phases. Orange dots represent the corresponding offline measurements.

In the next step, the hybrid approach was tested on an unseen dataset (process 3). All UKF parameters were retained from the previous setup, and the initial values for the state variables were identical for both the UKF and the CGM. The results of the UKF and the CGM are shown in Figure 5.

For glycerol (Figure 5B), both the UKF and the CGM predicted a slightly faster decrease in concentration than was indicated by the first available measurement at 20 h process time, showing a concentration of 0 g L^−1^. At the beginning of the second phase, both models predicted a slight increase in glycerol concentration. Otherwise, the concentration remained close to 0 g L^−1^ for the rest of the process, which was consistently predicted by both the UKF and the CGM. Biomass (Figure 5A) predictions from the UKF showed some fluctuations during the first process phase. However, in the second phase, both the UKF and the CGM provided good approximations of the measured biomass concentration. From around 50 h process time onward, the UKF predicted a further increase in biomass in the third process phase, resulting in a final value that distinctly overestimated the measured concentration. In contrast, the CGM provided a more accurate prediction in this phase. The depletion of L-tyr (Figure 5E) during the first phase was again not well captured by either model. In the second phase, both overestimated L-tyr concentrations, although the UKF predictions were closer to the experimentally measured values. In the production phase, the UKF predicted a slower decline, estimating the concentration to reach nearly 0 g L^−1^ only after approximately 58 h, whereas measurements indicated depletion already at around 48 h. In contrast, L-phe (Figure 5C) was again well approximated by the UKF. The concentration increased more slowly in this process compared to the previous process (process 2) and reached a final concentration of approximately 23 g L^−1^. The CGM predicted a slightly faster product formation and a maximum concentration of around 21 g L^−1^, not approximating the measured data as accurately as the UKF. Acetate (Figure 5D) concentration was again poorly predicted by both models. The CGM overestimated acetate production compared to the measured data, while the UKF underestimated the final acetate concentration.

**Figure 6:**
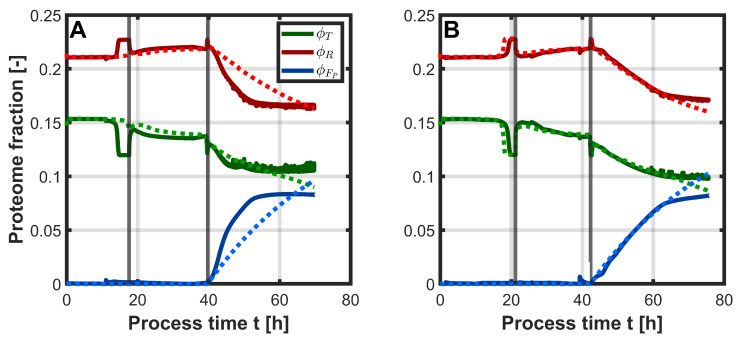
Time course of the proteome fractions for transport and catabolism ($\phi_{T}$, green), ribosomes ($\phi_{R}$, red), and L-phe production proteins ($\phi_{F_{p}}$, blue) over the course of the process. The fraction of the remaining proteins ($\phi_{Q}$) is not shown. Process 2 (**A**) can be seen on the left, and process 3 (**B**) can be seen on the right. Dashed lines represent predictions from the CGM, while solid lines show predictions from the UKF. Gray vertical lines indicate the different process phases.

In addition to extracellular concentrations, intracellular state variables are also considered in the CGM. One of these variables is the total protein content, which can be further divided into the individual proteome fractions. Figure 6 shows the proteome fractions for transport and catabolism ($\phi_{T}$), ribosomes ($\phi_{R}$), and L-phe production proteins ($\phi_{F_{p}}$) over the course of the process. Figure 6A displays the results for process 2, while Figure 6B shows the results for process 3. For process 2, a decrease in $\phi_{T}$ and a corresponding increase in $\phi_{R}$ can be observed toward the end of the batch phase in the UKF prediction, while the CGM simulation shows a relatively constant proteome distribution. This shift was likely caused by substrate depletion. During the second phase of the process, the proteome fractions remained relatively stable in both predictions. Upon induction in the third phase, $\phi_{F_{p}}$ increased, while both $\phi_{T}$ and $\phi_{R}$ decreased in both the CGM and UKF simulations. Interestingly, the increase in $\phi_{F_{p}}$ occurred more rapidly in the UKF, reaching its maximum of approximately 0.08 after just 50 h of process time. Additionally, the decline in $\phi_{R}$ was more pronounced than that of $\phi_{T}$. The UKF accurately predicted the L-phe production in process 2 (Figure 4C), particularly the rapid increase and the peak concentration toward the end of the process. Since the reaction rates in the CGM are coupled to the proteome fractions, this may explain the faster increase in the production-related proteome fraction observed in the UKF. For process 3, a relatively similar trend of the proteome fractions was observed between the UKF and CGM simulations. Only toward the end of the process did the UKF predict a slightly lower maximum for $\phi_{F_{p}}$ compared to the CGM. As the predicted L-phe concentrations in process 3 were closer between the UKF and CGM, this could explain the similar course of the proteome fractions.

For both process 2 and process 3, it was shown that the filter estimated some state variables more accurately than others. For example, substrate consumption in the batch phase was predicted to occur slightly too fast compared to the offline measurements. A closer look at the substrate concentration profile shows that it closely followed the PLSR prediction (Figure 3B). This can be explained by the initialization of the covariance matrix $P_{0}$ and the diagonal entries of the measurement noise matrix *R*. The diagonal entries of *R* are defined by the RMSE values from the PLSR results. Since the RMSEs for process 2 were calculated with offline measurements, the amount and time points of the measurements are relevant. As glycerol was almost immediately taken up by the cells after the batch phase, the offline measurements for glycerol after the batch phase were typically close to 0 g L^−1^. Higher glycerol concentrations were only observed during the batch phase, which were initially around 4 g L^−1^ and then decreased continuously. However, due to the lack of offline measurements during the batch phase, this phase did not contribute to the RMSE calculation. As a result, the RMSE for glycerol was underestimated, since it was calculated over a concentration range typically close to 0 g L^−1^. With more offline data during the batch phase, a more accurate RMSE estimate and, consequently, more realistic values in the diagonal of *R* might have been possible. Additionally, since the initial substrate concentrations varied slightly between processes, the corresponding diagonal entry in $P_{0}$ for glycerol was relatively large, causing the filter to rely more heavily on the measurement input. In the case of biomass, the UKF tended to overestimate, particularly toward the end of the processes. This again reflects the PLSR performance, which also consistently overestimated the biomass in both processes (Figure 3A). Nevertheless, the filter was able to compensate for this to some extent, resulting in relatively good approximations during the first two process phases. L-tyr concentrations were poorly estimated by both the UKF and the CGM. In both processes, its concentration remained consistently low. Since PLSR already overestimated L-tyr, this also carried over into the UKF predictions. A similar pattern was observed for acetate, which was also not well captured by the PLSR. As the underlying cause of acetate formation in the process is not entirely known, it is not well represented in the model, leading to poor predictions from both the UKF and the CGM. In contrast, the changes in L-phe concentration were predicted distinctly better with the UKF in both processes compared to the CGM alone. As the mechanism of product formation is also not as clear and, therefore, not easy to describe mathematically, it is represented in a simplified manner within the CGM, which limits its predictive capability under varying process conditions. However, since the PLSR accurately approximated the product concentration, the UKF was able to correct the CGM’s predictions accordingly.

## 4. Discussion

This study demonstrated that even a limited set of online process data can be sufficient to estimate important offline key variables with reasonable accuracy using PLSR. By integrating these predictions as measurements into a UKF with a CGM as the state estimator, the prediction quality could be further improved, providing further insights into intracellular states.

The training data from process 1 (reference process) was well approximated by the PLS models. However, the prediction performance for unseen process data (processes 2 and 3) was distinctly poorer. This discrepancy between well-approximated training data and less accurate predictions for unseen datasets is a common phenomenon for data-driven soft sensors, particularly in the context of biological processes [7,31]. In such cases, the model tends to overfit the training data, which often occurs if the training datasets are too small or not enough training data points are used. Another factor contributing to the reduced prediction accuracy is the inherent biological variability, as well as unexpected process disturbances. For example, in microbial processes, as is the case here, foam formation can occur. To counteract this, antifoam agents are added, which can influence oxygen transfer and, consequently, the pO_2_ in the bioreactor [32]. If the pO_2_ drops, the agitation speed and aeration rate are typically increased to maintain the defined pO_2_ setpoint. If these process variables are used as input variables for the PLSR, such abrupt changes can result in poor predictions. One approach for generating a more robust model could be to train it on data from multiple processes instead of only one [7]. This could improve model robustness and allow the model to better capture the biological and process variability present in different datasets.

One advantage of PLSR is its ease of implementation and its suitability for multicollinear and high-dimensional datasets. However, a disadvantage of PLSR is that, due to its linear structure, it often fails to fully capture highly nonlinear biochemical reaction systems. As an alternative to multivariate regression techniques such as PLSR, ANNs can be employed in data-driven approaches. Based on their structure, ANNs are capable of more effectively modeling complex nonlinear relationships, which are frequently encountered in biological processes [9]. Comparisons in the literature between various data-driven approaches such as PLSR, SVR, ANNs, and other multivariate regression methods for the prediction and monitoring of bioprocesses have demonstrated the superior performance of ANNs over PLSR and other regression techniques [11,33]. An additional strategy to enhance the predictive capabilities of the data-driven approach is to incorporate further measurements, thereby increasing the number of input variables. For instance, Lee et al. [34] demonstrated that L-phe and acetate spectra can be measured in situ by using Raman spectroscopy. These spectra could serve as additional input variables, potentially enabling more accurate and robust predictions. Alternatively, NIR spectroscopy could also be used for in situ determination of acetate and L-phe spectra [35]. In addition to the measurement of extracellular variables, intracellular quantities such as fluorescent proteins can also be measured and used as input variables. The strain used in this study expresses four different fluorescent proteins under various physiological conditions, intended to reflect specific cellular states such as growth, oxygen availability, general stress, or product formation [25]. In particular, growth- and product formation-related fluorescence may represent valuable information that correlates with biomass and product concentrations. One way to monitor these signals at-line could be through an automated sampling system coupled with a flow cytometer, which is also planned as future work. This approach is also referred to as automated real-time flow cytometry (ART-FCM) in the literature and has already been employed for the monitoring of bioprocesses, for instance, to observe product formation or cellular stress responses [36,37]. The measurement of these intracellular variables offers the additional advantage that they can not only be correlated with extracellular process variables but also be directly integrated into the CGM as measurements in the hybrid modeling approach.

For the hybrid UKF approach, several challenges remain. Two of the most important parameters for the KF are the process and measurement noise covariance matrices *Q* and *R* [38]. In practical applications with real sensor or measurement data, the measurement noise *R* is often more straightforward to estimate. However, in this approach, where measurements are derived from PLS models, estimating *R* is more challenging. Here, the diagonal entries of *R* were determined based on the MSE between the PLSR predictions and the offline measurements. However, if there are insufficient offline measurements available to reliably estimate the MSE, this can lead to poor correction performance by the filter. As previously mentioned, no offline measurements are available during the batch phase, resulting in an unrealistically low MSE for glycerol, since the concentration remains near zero after the batch phase. Another limitation is that both *R* and *Q* are kept constant throughout the entire process. Given that the process is divided into three distinct phases, each characterized by different system dynamics, the prediction accuracy of the PLS models also varies across each phase. A straightforward improvement would be to define the measurement noise matrices *R* for each phase. However, due to the lack of offline data in the batch phase, the measurement values would need to be estimated or calculated through simulations. Similarly, the process noise *Q* is likely to change over time. Here also, the process noise for each phase or even dynamic *Q* could improve correction performance. One way to dynamically estimate *Q* is to incorporate parameter uncertainty. As shown by Kraemer and King [39] and Tuveri et al. [28], the process noise covariance matrix $Q_{k}$ can be defined as

(26) $$Q_{k}=G_{k}\;Q_{w}\;G_{k}^{T}$$

where $G_{k}$ is the Jacobian of the model with respect to the parameters and $Q_{w}$ is the parameter covariance matrix. The lower bound of $Q_{w}$ can be approximated using the Fisher Information Matrix, although it represents only a lower bound, and the true variance may be higher. An alternative strategy that could be used for dynamically updating *R* and *Q* is based on innovation analysis. Zheng et al. [40] proposed to evaluate the innovations using a chi-squared distributed test statistic and, if necessary, recalculate the measurement and process noise covariance matrices *R* and *Q* based on the residuals and innovations, as well as the previous estimates of *Q* and *R*. This has the advantage that not much knowledge for the determination of *Q* and *R* is necessary.

Another aspect that should be considered is the observability of the system, which indicates whether the system states can be reconstructed solely based on the available measurements. Particularly for future work, the observability of the CGM should be investigated to determine which variables must be measured to fully reconstruct the system. Dewasme et al. [41] showed that for an *E. coli* fermentation, measurements with only a biomass probe were sufficient to describe both acetate and substrate concentrations. This would also open up the possibility of only using a single biomass probe with a UKF; therefore, the correction would be less reliant on the accuracy of the PLSR estimations. Nevertheless, for industrial-scale processes, such probes are generally less suitable, as they provide only local measurements and may not accurately represent the entire reactor [11]. Also, when only using a biomass probe, an accurate model capable of reliably representing the system states is necessary. This is also an additional challenge that is evident in the results presented here. If the dynamic behavior of the system is poorly represented by the state estimator, the UKF can only partially compensate for the discrepancy. Similarly, this can also be seen for the measurements. If the PLS models do not accurately reflect the true offline values, the UKF can only correct based on the limited information it receives. This is evident in the case of acetate, where neither the CGM nor the PLS models can adequately capture the concentration profile, and, consequently, the UKF also fails to provide accurate estimates. Nevertheless, relatively simple models combined with an EKF or a UKF can be sufficient to achieve adequate state estimation, provided that the models are sufficiently parameterized. Additionally, kinetic parameters exhibiting high sensitivity can also be estimated within the filter framework to improve state estimation accuracy [13,41,42]. To achieve this, sensitive parameters can be identified through a sensitivity analysis and can then be incorporated into the extended state vector for estimation [43]. This aspect should also be taken into account in future work using CGMs, as kinetic parameters often need to be estimated via regression from experimental data [20]. This was also the case in the present study, where parameter values were obtained through least-squares minimization (see Supplementary Materials).

Another interesting result is the correction of the proteome fractions in the UKF. Since intracellular states, such as metabolite and protein concentrations, are not measured directly, the proteome fractions can only be estimated indirectly. Particularly interesting is the correction of the proteome term for the production proteins $\phi_{F_{p}}$. For process 2, 0.01 mM IPTG instead of 0.3 mM was used for induction, which surprisingly led to higher measured concentrations of L-phe. In fact, a lower production would be expected, as fewer production proteins should be expressed. One possible explanation for the improved production could be a reduced metabolic burden due to weaker induction. The strain harbors a medium-high copy plasmid with a strong promoter [44], and therefore, a full induction may force the cell to use more resources for protein synthesis, leaving fewer resources for product formation [45]. Since the effect of the inducer concentration is not included in the CGM, this effect cannot be explained by the model. Here, more experimental information is necessary, for example, measurements of the proteome during the production phase, to include this effect. Currently, the L-phe production rate increases linearly with the proteome fraction $\phi_{F_{p}}$. However, a rate-limiting step might occur earlier in the metabolism, which could impose an upper limit on the amount of production proteins actually required to achieve maximum product formation. By including such an upper limit in the CGM, a more realistic description of the system might be possible.

By incorporating additional input variables through ART-FCM and using adapted or dynamic measurement and process noise covariance matrices, prediction accuracy could be improved. This, in turn, would allow the CGM to provide more detailed and reliable information about the process and the intracellular state variables.

## 5. Conclusions

Real-time information on process variables is essential for process optimization and control in biotechnological processes. In addition to extracellular variables such as biomass, substrate, or product concentrations, intracellular factors such as protein concentration and distribution play an important role in ensuring optimal product formation. In this study, a hybrid modeling approach combining PLSR and a UKF was applied to estimate the state variables, namely biomass, glycerol, L-phe, acetate, and L-tyr, in an L-phe production process using a recombinant *E. coli* strain. The PLS models alone achieved accurate predictions for L-phe; moderate performance for biomass, glycerol, and L-tyr; and limited accuracy for acetate. Incorporating these predictions as measurements into a UKF, in which a CGM was used as the state estimator, significantly improved the estimation of L-phe concentrations compared to the CGM alone, while also enabling the estimation of intracellular variables.

However, further improvement is needed for the reliable estimation of the remaining state variables. To achieve this, future work will focus on improving the data-driven model predictions with additional real-time information. This will be achieved through the use of the four integrated fluorescent proteins in the *E. coli* strain used here in combination with ART-FCM. Additionally, ANNs will be applied to evaluate prediction accuracy compared to PLSR. Also, the CGM will be improved by incorporating real proteome data from the process, with the goal of gaining a better understanding of the mechanisms for product and byproduct formation.

## Figures and Tables

**Table 1:** RMSEs of the state variables (biomass, glycerol, L-phe, acetate, and L-tyr) from PLSR training and prediction. The RMSEs for process 1 were calculated based on the model predictions and training data, while for processes 2 and 3, the RMSEs were computed using offline measurements and the interpolated model predictions.

State Variables	RMSE		
	**Process 1 (Training)**	**Process 2 (Prediction)**	**Process 3 (Prediction)**
Biomass [g L^−1^]	0.95	5.59	5.8
Glycerol [g L^−1^]	0.12	0.24	0.22
L-phenylalanine [g L^−1^]	0.43	1.81	0.99
Acetate [g L^−1^]	0.3	1.29	1.61
L-tyrosine [g L^−1^]	0.02	0.09	0.04

**Table 2:** RMSEs of the state variables (biomass, glycerol, L-phe, acetate, and L-tyr) of the CGM and the UKF for processes 2 and 3. The RMSEs were calculated based on the offline measurements and the interpolated model predictions.

State Variables	RMSE			
	**Process 2**		**Process 3**	
	**CGM**	**UKF**	**CGM**	**UKF**
Biomass [g L^−1^]	3.15	1.94	1.74	3.43
Glycerol [g L^−1^]	0.15	0.19	0.18	0.2
L-phenylalanine [g L^−1^]	5.14	0.76	2.48	0.87
Acetate [g L^−1^]	3.51	1.47	3.84	1.51
L-tyrosine [g L^−1^]	0.07	0.1	0.06	0.03

## Data Availability

The raw data supporting the conclusions of this article will be made available by the authors on request.

## Supplementary Materials

The following supporting information can be downloaded at https://www.mdpi.com/article/10.3390/bioengineering12060654/s1, Table S1: Estimated parameters for the CGM (process 1), along with their values, units, and standard deviations. Figure S1. Simulation of process 1 with the CGM and the optimal parameter values determined by least-squares minimization using offline measurements. The CGM simulations are shown as blue solid lines, while the offline measurements are represented by orange dots for the state variables: biomass (A), glycerol (B), L-phe (C), acetate (D), and L-tyr (E). The error bars indicate the standard error from the parameter estimation. Figure S2. Simulation of process 1 with the CGM and the optimal parameter values determined by least-squares minimization using offline measurements. The CGM simulations are shown as blue solid lines, while the offline measurements are represented by orange dots for the state variables: biomass (A), glycerol (B), L-phe (C), acetate (D), and L-tyr (E). The light-blue areas represent the 95% confidence intervals of the model predictions. Figure S3. Process data of process 1: pO_2_ (A), stirrer (B), air flow (C), off-gas CO_2_ (D), substrate feed (E), pH (F), temperature (G), and base addition (H). Figure S4. Process data of process 2: pO_2_ (A), stirrer (B), air flow (C), off-gas CO_2_ (D), substrate feed (E), pH (F), temperature (G), and base addition (H). Figure S5. Process data of process 3: pO_2_ (A), stirrer (B), air flow (C), off-gas CO_2_ (D), substrate feed (E), pH (F), temperature (G), and base addition (H). Figure S6. Cross-validation with k = 5 for the batch phase (A), biomass production phase (B), and production phase (C) using the training data from process 1 (reference process). Figure S7. Predictions using the trained PLS models on the unseen dataset from process 3 with online process variables as inputs. Vertical gray lines indicate the different process phases. Model predictions for the state variables (biomass (A), glycerol (B), L-phe (C), acetate (D), and L-tyr (E)) are shown as black dotted lines over process time. Orange dots represent the corresponding offline measurements. References [20,46] is cited in the Supplementary Materials.
