# Supplementary material for: A Hybrid Soft Sensor Approach Combining Partial Least-Squares Regression and an Unscented Kalman Filter for State Estimation in Bioprocesses
Source: Bioengineering (Basel). 2025 Jun 15;12(6):654. doi: 10.3390/bioengineering12060654 (PMC12189592; doi:10.3390/bioengineering12060654)
Supplement: Supplementary file 1 [file bioengineering-12-00654-s001.zip › bioengineering-3618622-supplementary.pdf]

Supplementary Materials: A hybrid soft sensor approach combining partial least squares regression and an unscented Kalman filter for state estimation in bioprocesses

Lucas Hermann [1] and Andreas Kremling [1,*]

1. The coarse-grained model

The coarse-grained model (CGM) was adapted from Doan et al. [1]. The model describes the following state variables: volume V, glycerol S, biomass X, L-phenylalanine (L-phe) F, acetate O, and L-tyrosine (L-tyr) Tyr. It also describes the following intracellular state variables: metabolites M, proteins P, residual biomass U, and L-phenylalanine production proteins ϕ_{F_p}. The differential equations for the state variables are as follows:

$$
\begin{aligned}
\dot{V} &= q_{in} \\
\dot{S} &= \frac{q_{in}}{V}\,(S_{in} - S) - r_T\,X\,\omega_S \\
\dot{X} &= \mu\,X - \frac{q_{in}}{V}\,X \\
\dot{M} &= \underline{n}_M^T\,\underline{r} - \mu\,M \\
\dot{P} &= \underline{n}_P^T\,\underline{r} - \mu\,P \\
\dot{U} &= \underline{n}_U^T\,\underline{r} - \mu\,U \\
\dot{F} &= \frac{q_{in}}{V}\,(F_{in} - F) - (\mu\,\frac{Y_{X,F}}{\omega_F} - r_F)\,X\,\omega_F \\
\dot{O} &= r_O\,X\,\omega_O - \frac{q_{in}}{V}\,O \\
\dot{Tyr} &= \frac{q_{in}}{V}\,(Tyr_{in} - Tyr) - \mu\,Y_{X,Tyr}\,X \\
\dot{\phi_{F_p}} &= \frac{IPTG}{IPTG + K_{IPTG}}\,k_{trans} - k_{deg}\,\phi_{F_p}
\end{aligned}
\tag{1}
$$

In the equations, q_{in} is the volume inflow; S_{in}, F_{in}, and Tyr_{in} are the respective glycerol, L-phe, and L-tyr concentrations in the feed; r_i represents the rates, which are given in 2; ω_i is the respective molecular weight; μ is the growth rate; n_i is the respective row in the stoichiometric matrix N (4); and $Y_{X,i}$ represents the yield coefficients. In $\dot{\phi_{F_p}}$, all variables are constant parameters, except for ϕ_{F_p}.

The rates are given in 2, and the proteome fractions T, R, Q, and F_p are given in 3:

$$
\begin{aligned}
r_T &= k_T\,\frac{S}{S + K_T}\,T \\
r_P &= k_P\,\frac{M}{M + K_P}\,R\,(0.1 + (0.9\frac{Tyr}{Tyr + K_{Tyr}})) \\
r_O &= k_O\,M\,T\,\frac{1}{1 + \exp(K(F_P - F_{P,H}))} \\
r_U &= k_U\,\frac{M}{M + K_U}\,R\,(0.1 + (0.9\frac{Tyr}{Tyr + K_{Tyr}})) \\
r_F &= k_F\,M\,F_P\,\frac{1}{1 + \frac{O}{K_{I,O}}} \\
r_C &= k_C\,M\,T
\end{aligned}
\tag{2}
$$

All k_i and K_i are constant parameters, determined by fitting to the experimental data. The parameters T_m, T_l, R_m, and R_l are determined by correlating the proteome data from the literature with the intracellular metabolite concentrations, as described in [1].

$$\begin{aligned} T &= ((T_m\, C + T_l)\; P)(1 - \frac{\phi_{F_p}}{T_l + R_l}) \\ R &= ((R_m\, C + R_l)\; P)(1 - \frac{\phi_{F_p}}{T_l + R_l}) \\ Q &= 1 - \frac{T + R}{P} + \phi_{F_p} \\ F_P &= \phi_{F_p}\; P \end{aligned} \tag{3}$$

The stoichiometric matrix N is given in 4, and the growth rate is given in 1:

$$N = \begin{pmatrix} \alpha & -\gamma & -0.65 & -\delta & -1.8 & -0.48 \\ 0 & \beta & 0 & 0 & 0 & 0 \\ 0 & 0 & 0 & \varepsilon & 0 & 0 \end{pmatrix} \tag{4}$$

$$\mu = \underline{\omega}^T\; N\; \underline{r} \tag{5}$$

1.1. Parameter estimation

The parameters were adapted from [1]. With slight changes to the initial model and a new process, the parameters were determined by minimizing the least squares between the model and the offline measurements of process 1. Additionally, the standard deviation of the estimated parameters was calculated. For this purpose, the Fisher Information Matrix (FIM) was computed and used as a lower bound for the parameter covariance matrix [2]. The FIM can be calculated as follows:

$$FIM_p = n(RSS)^{-1} J\; J^T\big|_{p=\hat{p}} \tag{6}$$

where n denotes the total number of samples, RSS is the weighted residual sum of squares, and J represents the Jacobian matrix of the residuals with respect to the optimal parameters $(p = \hat{p})$. The parameter covariance matrix can then be calculated using the FIM:

$$cov(\hat{p}) = FIM_p^{-1} \tag{7}$$

All estimated parameters, along with their values, units, and standard deviations, are shown in Table S1.

Table S1. Estimated parameters for the CGM (process 1), along with their values, units, and standard deviations.

Parameter	Value	Unit	Std. Dev.
k_T	$2.97 \cdot 10^3$	h^{-1}	$4.53 \cdot 10^2$
k_P	2.42	h^{-1}	0.67
k_O	$2 \cdot 10^{10}$	$g\,mol^{-1}\,h^{-1}$	$5.39 \cdot 10^9$
k_U	0.42	h^{-1}	0.11
k_F	$3.36 \cdot 10^8$	$g\,mol^{-1}\,h^{-1}$	$1.37 \cdot 10^8$
k_C	$6.04 \cdot 10^8$	$g\,mol^{-1}\,h^{-1}$	$7.49 \cdot 10^7$
K_T	$7.33 \cdot 10^{-6}$	$g\,L^{-1}$	$1.45 \cdot 10^{-6}$
K_P	$9.28 \cdot 10^{-2}$	$mol\,g^{-1}$	$3.18 \cdot 10^{-2}$
K_{Tyr}	$8.97 \cdot 10^{-6}$	$mol\,g^{-1}$	$2.19 \cdot 10^{-6}$
K_U	$5 \cdot 10^{-5}$	$mol\,g^{-1}$	$2.05 \cdot 10^{-5}$
$K_{i,O}$	1.05	$mol\,g^{-1}$	0.31
k_{trans}	$5 \cdot 10^{-3}$	-	$1.81 \cdot 10^{-3}$
k_{deg}	$2.83 \cdot 10^{-2}$	-	$7.05 \cdot 10^{-3}$
K	-124	-	58
$F_{P,H}$	0.12	-	$4.2 \cdot 10^{-2}$

Since glycerol, L-phe, acetate, and L-tyr were only measured once, the error variance was additionally calculated:

$$\hat{\sigma}^2 = \frac{\sum_{i=1}^{n} (y_i - \hat{y}_i)^2}{n - p} \quad (8)$$

where y_i represents the measured value, $\hat{y}_i$ is the model prediction, n is the number of samples, and p is the number of estimated parameters. The square root of the resulting error variance was then taken to represent the standard error, which was then used to display the error bars of the measurements, as shown in Figure S1. In addition to the standard deviations of the estimated parameters and the standard error, the 95% confidence intervals of the CGM predictions were calculated:

$$f(p) \pm t_{n-p,1-\alpha/2}\, \hat{\sigma} \sqrt{a^\top \left(J^\top J\right)^{-1} a} \quad (9)$$

where a describes how the states change with respect to the parameters p.

$$a = \frac{\partial f(x,p)}{\partial p} \quad (10)$$

The resulting confidence intervals for each simulated state can be seen in Figure S2.

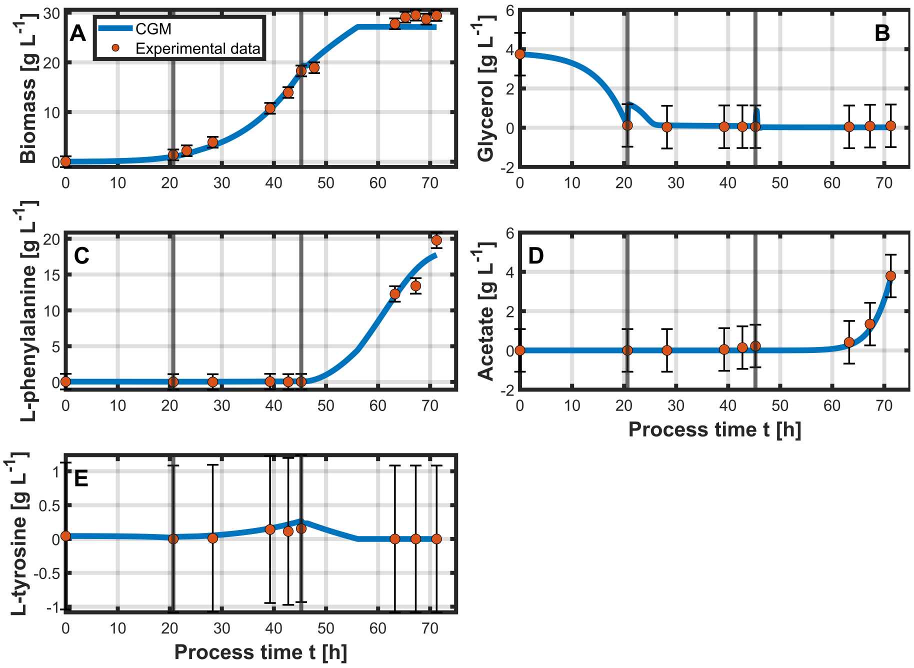

Figure S1. Simulation of process 1 with the CGM and the optimal parameter values determined by least-squares minimization using offline measurements. The CGM simulations are shown as blue solid lines, while the offline measurements are represented by orange dots for the state variables: biomass (**A**), glycerol (**B**), L-phe (**C**), acetate (**D**), and L-tyr (**E**). The error bars indicate the standard error from the parameter estimation.

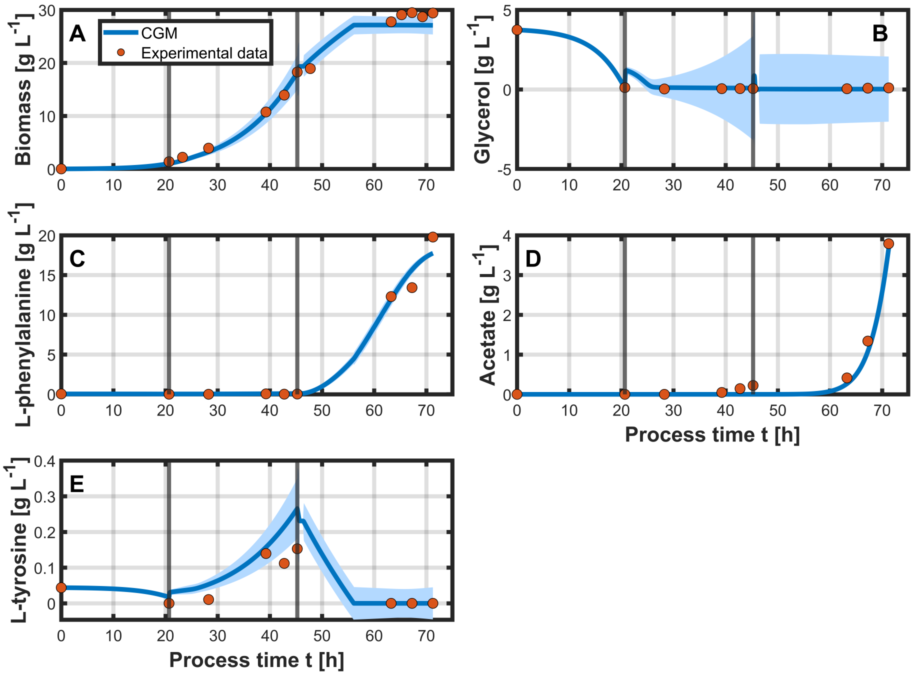

Figure S2. Simulation of process 1 with the CGM and the optimal parameter values determined by least-squares minimization using offline measurements. The CGM simulations are shown as blue solid lines, while the offline measurements are represented by orange dots for the state variables: biomass (**A**), glycerol (**B**), L-phe (**C**), acetate (**D**), and L-tyr (**E**). The light-blue areas represent the 95% confidence intervals of the model predictions.

2. Online process data

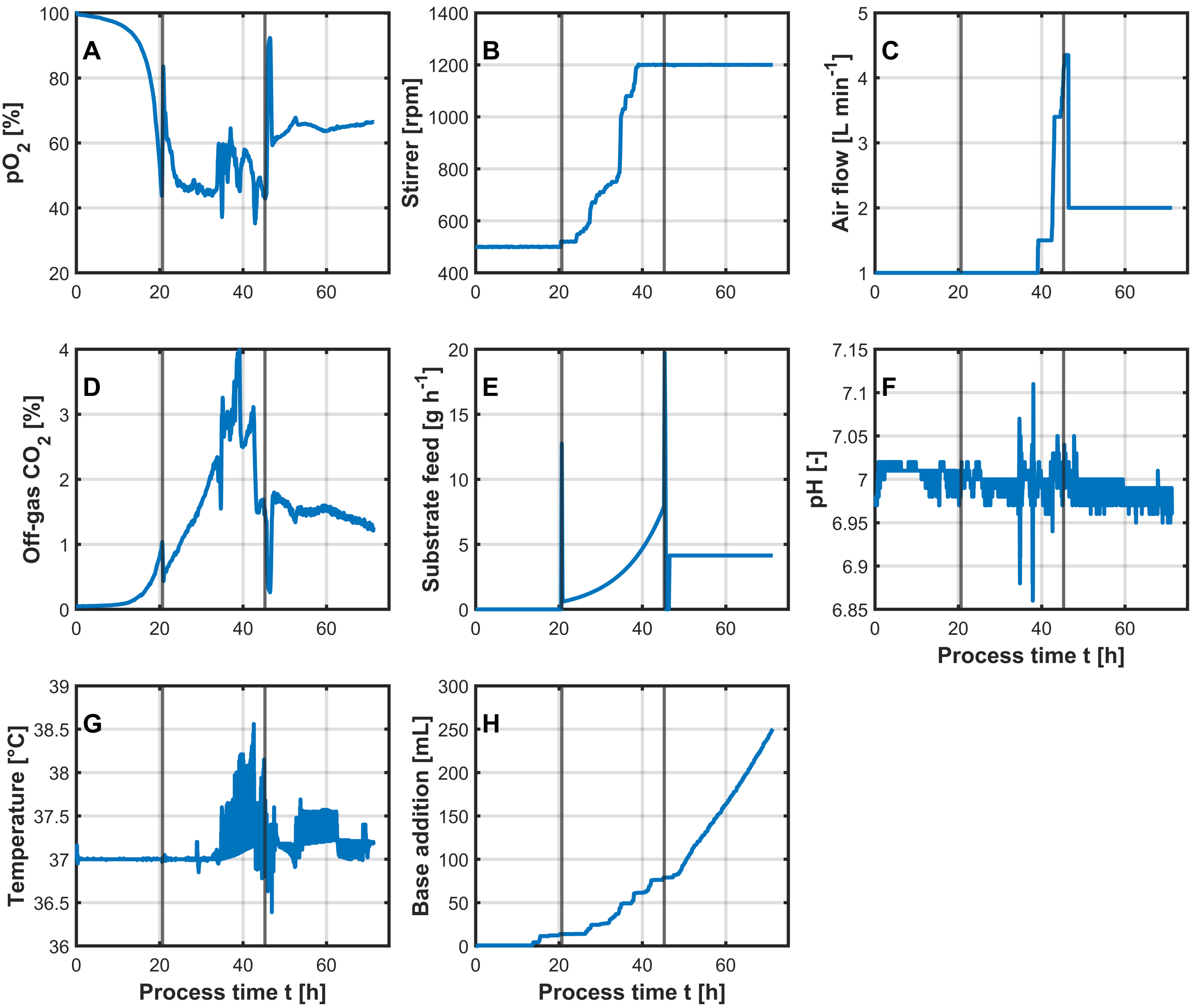

Figure S3. Process data of process 1: pO_2 (A), stirrer (B), air flow (C), off-gas CO_2 (D), substrate feed (E), pH (F), temperature (G), and base addition (H).

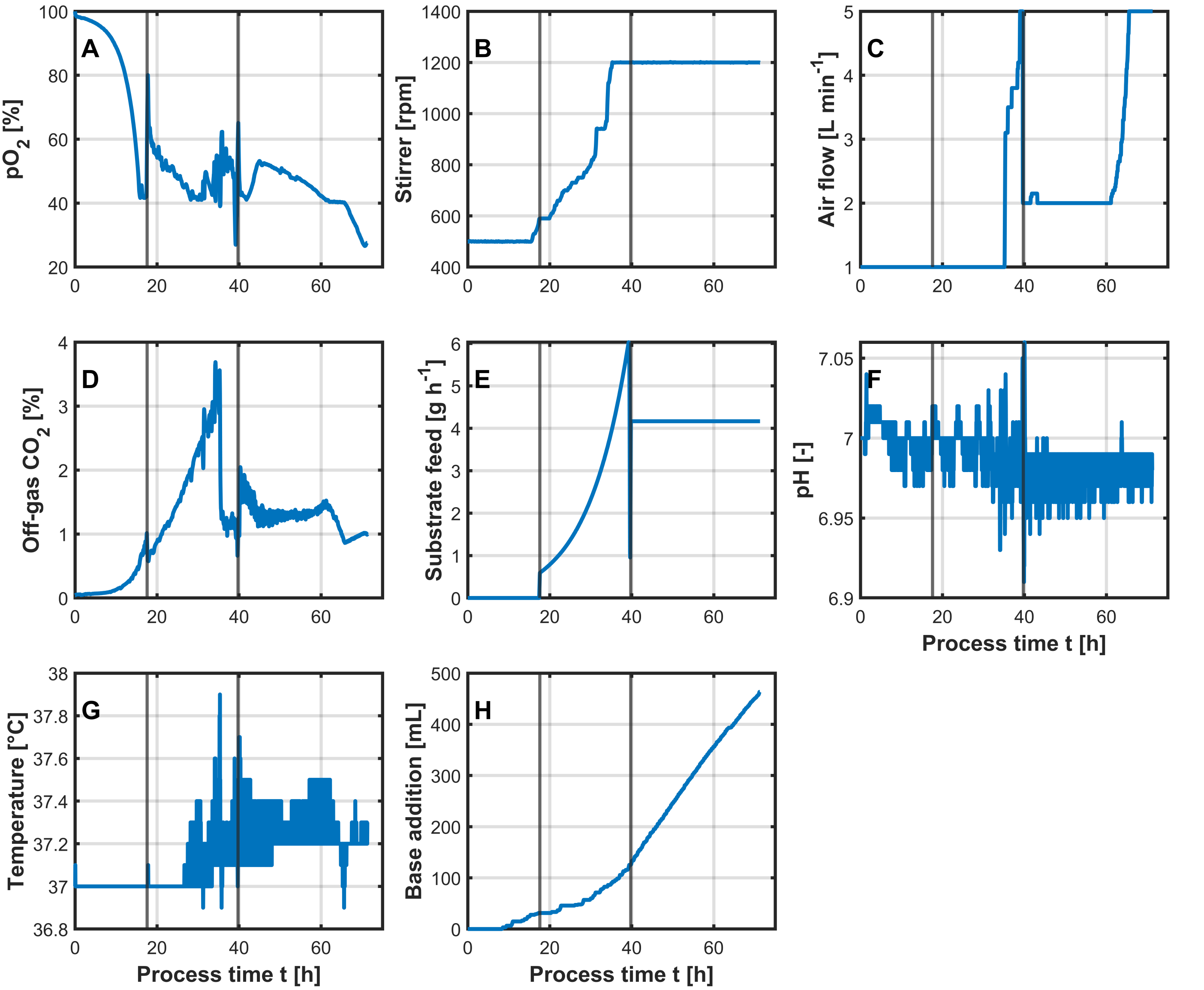

Figure S4. Process data of process 2: pO_2 (A), stirrer (B), air flow (C), off-gas CO_2 (D), substrate feed (E), pH (F), temperature (G), and base addition (H).

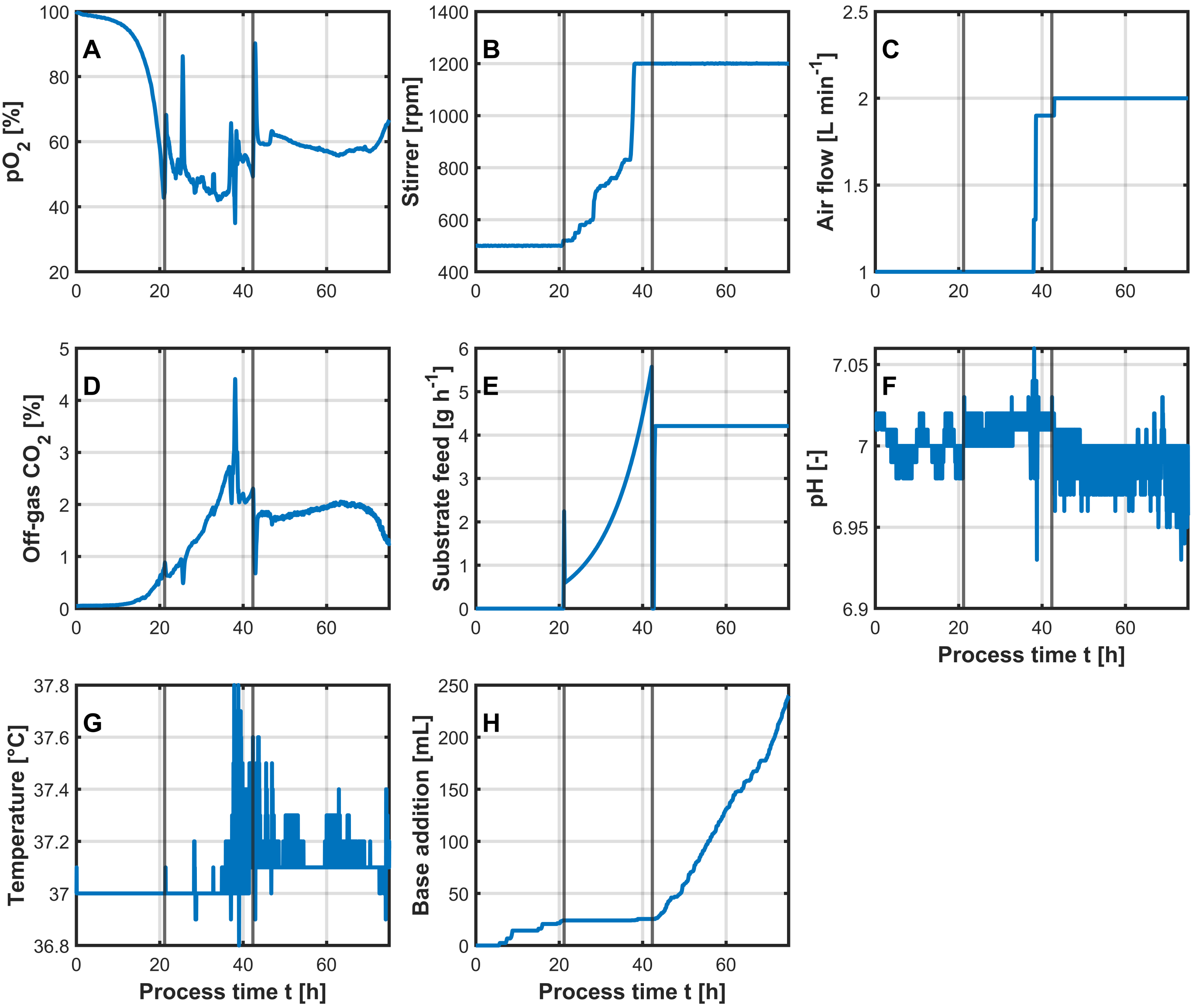

Figure S5. Process data of process 3: pO_2 (A), stirrer (B), air flow (C), off-gas CO_2 (D), substrate feed (E), pH (F), temperature (G), and base addition (H).

3. PLSR

3.1. Cross-validation

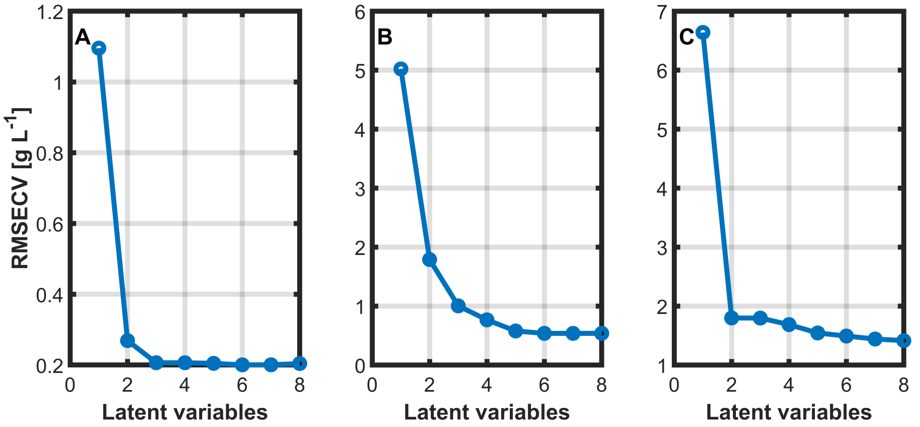

Figure S6. Cross-validation with k = 5 for the batch phase (A), biomass production phase (B), and production phase (C) using the training data from process 1 (reference process).

3.2. Prediction process 3

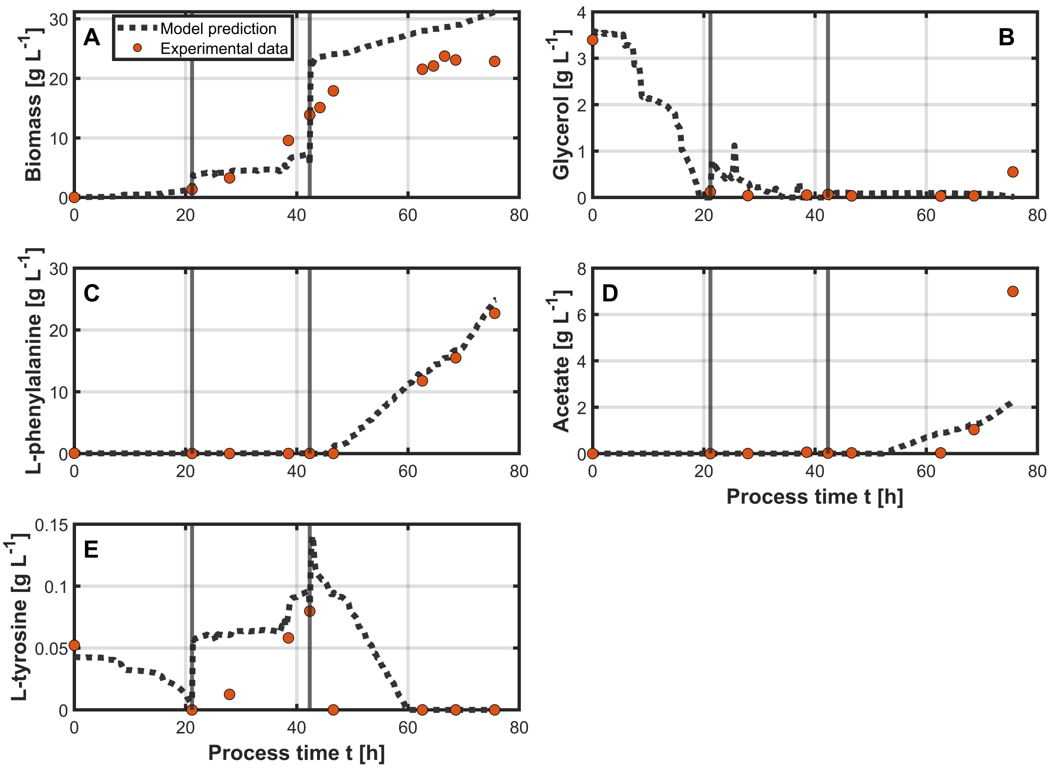

Figure S7. Predictions using the trained PLS models on the unseen dataset from process 3 with online process variables as inputs. Vertical gray lines indicate the different process phases. Model predictions for the state variables (biomass (A), glycerol (B), L-phe (C), acetate (D), and L-tyr (E)) are shown as black dotted lines over process time. Orange dots represent the corresponding offline measurements.

References

1. Doan, D.T.; Hoang, M.D.; Heins, A.L.; Kremling, A. Applications of Coarse-Grained Models in Metabolic Engineering. *Front. Mol. Biosci.* **2022**, *9*, 806213.
2. van Riel, N.A.W. A template for parameter estimation with Matlab Optimization Toolbox; including dynamic systems. Available online: www.researchgate.net/publication/269930829_A_template_for_parameter_estimation_with_Matlab_Optimization_Toolbox_including_dynamic_systems (accessed on 11 June 2025).
